# Editorial: Live Biotherapeutic Products: where are we?

**DOI:** 10.3389/frmbi.2025.1664282

**Published:** 2025-09-15

**Authors:** Glenn Tillotson

**Affiliations:** Independent researcher, North, VA, United States

**Keywords:** Live Biotherapeutic Products, fecal transplantation, safety, *Clostridium difficile*, regulatory procedure

In the fourth century BCE, Ge Hong recognized the utility of “yellow soup”, a fecally originated product in managing diarrheal disease (Zhang et al., 2012). In Europe, the first recorded use of Fecal Microbiome Transplantation (FMT) took place in veterinary medicine. The procedure was carried out by Fabricius Acquapendente (1537–1619), an Italian anatomist and surgeon, who transferred gastrointestinal contents from a healthy animal to a sick one. This treatment, known as “transfaunation”, became a widespread treatment for animals and was used for restoring normal rumination in cattle (Borody et al., 2004). Interest in the use of the restoration of the microbiome as a means of treating *C. difficile* infection (CDI) recurrences has re-ignited in the past decade. In this Research Topic, various aspects of this management process are discussed from a variety of perspectives.

The FDA’s approval of Rebyota and Vowst in 2022 and 2023, respectively, was considered a landmark in terms of the development of novel therapies for treating recurrent *Clostridiodes difficile* infections. Navakelke and Chopra discussed the potential of Live Biotherapeutic Products (LBP) for human conditions, acknowledging that we have reached a turning point in recognizing the truly amazing breadth of conditions that can be managed.

Prior to the approval of these two LBPs there were significant challenges posed by regulators in terms of guidance and analytical frameworks. These were elegantly highlighted by the Microbiome Therapeutics Innovation Group (MITG) and Barberio. It was clear that existing regulatory frameworks for governing LBPs had significant gaps. These included microbial identification, potency and bioburden. The MITG led collaborative efforts to engage experts in the field to hold discussions with the FDA. These meetings discussed catalyzing improvements in LBP analytics and refreshing the regulatory landscape. It was clear that a multi-faceted approach to the continued development of LBPs is essential.

Slijkerman et al. discussed the challenges faced when expanding LBP development while ensuring product safety and effectiveness. Previous recent experiences with non-regulated fecal materials for transplantation were associated with infections leading to some deaths notably among immunocompromised patients. Thus the focus shifted from traditional methods to developing safe products for use in fecal transplantation, namely live microbial products (LMPs). LMPs are subdivided into specific subcategories and their product characteristics led to the development of appropriate guidelines. LMPs are subject to GMP-compliant manufacturing guidance. However, there are no guidelines at present for injectable LMPs. Safety-related quality analysis is often inappropriate. Current FDA guidelines require screening for 29 pathogens to be absent from currently approved LBPs. This list is updated as necessary. Slijkerman et al. highlighted LMP guidelines and their different subcategories.

Thomas et al. presented the value of microbial consortia used in laboratory studies. Keratinocyte dysfunction is intrinsically involved in skin barrier repair and wound healing. Their study suggested that probiotic supplementation with two strains each of *Lactobacillus* and *Bifidobacterium* spp. may improve wound management. Interestingly the metabolome profile of these strains exerts a positive effect on key cell lines. These observations are encouraging for managing skin functionality.

Allergic conditions are a major global health problem. The role of the microbiome on immune function and many subsequent conditions in early life is becoming clearer. Tarrant and Finlay examined the potential of LBPs for the prevention and clinical management of childhood allergies. However, the present findings are inconsistent and somewhat limited. The authors discussed the current research and highlighted future possibilities. It has been shown that LBPs can mediate allergy susceptibility. These include several mechanisms such as Th1/Th2 balance, SCFA-induced inactivation of GPR41/43, and HDAC inhibition. It is these outcomes that suggest that LBPs may have value in managing allergic conditions.

Chronic conditions carry a significant burden due to elevated mortality and economic impact. Postbiotics are bioactive compounds that could be used as a possible therapeutic approach for chronic diseases. Asefa et al. discussed the potential of postbiotics in managing non-communicable diseases such as diabetes, cancer, obesity and certain cardiovascular conditions. The authors discussed the various mechanisms that could exert beneficial effects. These include immune response, immune modulation, and reduction of inflammation, leading to improved gut barrier function to improve permeability. A deeper understanding of these effects could enhance health outcomes in various at-risk populations.

LBPs have been extensively studied in *Clostridiodes difficile* infection (CDI). Sehgal and Feuerstadt discussed the effectiveness of LBPs in the treatment of recurrent CDI. Over the past decade our understanding of rCDI has shown that the relationship of dysbiosis (a disturbance of gastrointestinal microbial flora) provides an ideal setting for *C difficile* infection and for recurrent infections. Antibiotics have generally been effective in treating the causative infection, but can do nothing toward rebalancing the dysbiosis. The value of replacing lacking key species by using LBPs has shown the importance of reversing the microbial imbalances and developing eubiosis. The use of fecal microbiome therapy (FMT) has had variable success over the past decade. The introduction of healthy, human-derived feces into patients suffering from rCDI, as with the aforementioned products Rebyota and Vowst, has shown comparable effectiveness and safety. These are safe and effective when administered after standard antimicrobial therapy. These LBPs offer a new and safe approach to rCDI.

Lactobacillus is the dominant species in a healthy vagina and provides the first line of defense against pathogens. Vaginal dysbiosis is characterized by the loss of lactobacillus species, and is associated with genital diseases such as bacterial vaginosis, aerobic vaginosis, vulvo-candidiasis, sexually transmitted infections and pregnancy complications. Conventional treatments do not restore the protective flora of the vagina. Valeriano et al. examined the potential of LBPs in remedying dysbiosis. Lactobacillus species display a range of genotypic and phenotypic features, which include lactic acid production, inhibition of pathogens to epithelial cells, and other essential characteristics.

Consortia of specific organisms are being studied to develop Vaginal Microbiome Therapy (VMT). Valeriano et al. showed that these products are undergoing the standard range of FDA regulatory steps including the assessment of efficacy and tolerability. An appreciation of the many vaginal conditions and their causative organisms and disturbed environment is likely to improve the management of a range of conditions, which carry a significant morbidity and have significant financial implications with the use of VMT.

Finally, Navalkele and Chopra highlighted the application of LBPs in the management of different infectious diseases. As previously discussed, the microbiome consists of many microorganisms, including bacteria, fungi, viruses, and parasites. These organisms are found throughout the body. The most abundant location for these organisms is the gastrointestinal tract. The microbiome supports many bodily functions, as demonstrated by the well-established gut-brain axis. Dysbiosis is associated with immune-mediated conditions such as obesity, cancer and many others. The replacement of antibiotic resistant species found in the gastrointestinal tract by LBPs holds significant promise.

Perhaps the most relevant application of specific microbiome changes is in the management of COVID-19, a novel approach in which LBP LL-37 can prevent the binding of the virus to the host cell receptor ACE2 thus reducing the risk of infection. Other infections that are under investigation include those of the respiratory and urinary tracts, HIV, bacterial vaginosis and candidiasis. The status of the various clinical trials was summarized by these authors.

Overall, Live Biotherapeutic Products hold significant promise as therapeutic or preventative options for various human diseases and conditions. The development of these products is subject to evolving regulations, including those related to manufacturing processes and safety. As these become more defined, LBPs will build on the work of Ge Hong and his “yellow soup”.
